# Elevated CD21^low^ B Cell Frequency Is a Marker of Poor Immunity to Pfizer-BioNTech BNT162b2 mRNA Vaccine Against SARS-CoV-2 in Patients with Common Variable Immunodeficiency

**DOI:** 10.1007/s10875-022-01244-2

**Published:** 2022-03-15

**Authors:** Peter Bergman, David Wullimann, Yu Gao, Emilie Wahren Borgström, Anna-Carin Norlin, Sara Lind Enoksson, Soo Aleman, Hans-Gustaf Ljunggren, Marcus Buggert, C. I. Edvard Smith

**Affiliations:** 1 Department of Infectious Diseases, Immunodeficiency Unit, Karolinska University Hospital, Stockholm, Sweden; 2 Department of Laboratory Medicine, Clinical Microbiology, Karolinska Institutet, Stockholm, Sweden; 3 Department of Medicine Huddinge, Center for Infectious Medicine, Karolinska Institutet, Stockholm, Sweden; 4 Department of Clinical immunology and Transfusion Medicine, Karolinska University Hospital, Stockholm, Sweden; 5 Department of Clinical Science, Investigation and Technology (CLINTEC), Karolinska Institutet, Stockholm, Sweden; 6 Department of Medicine Huddinge, Infectious Diseases, Karolinska Institutet, Stockholm, Sweden; 7 Department of Laboratory Medicine, Biomolecular and Cellular Medicine, Karolinska Institutet, Stockholm, Sweden

**Keywords:** CD21^low^ B-cells, Pfizer-BioNTech BNT162b2 mRNA vaccine, COVID-19, SARS-CoV-2, CVID

## Abstract

**Purpose:**

Limited data is available on the effect of COVID-19 vaccination in immunocompromised individuals. Here, we provide the results from vaccinating a single-center cohort of patients with common variable immunodeficiency (CVID).

**Methods:**

In a prospective, open-label clinical trial, 50 patients with CVID and 90 age-matched healthy controls (HC) were analyzed for SARS-CoV-2 spike antibody (Ab) production after one or two doses of the Pfizer-BioNTech BNT162b2 mRNA vaccine. Additionally, in selected patients, SARS-CoV-2 spike-specific T-cells were assessed.

**Results:**

A potent vaccine-induced anti-spike–specific IgG Ab response was observed in all the HC. In contrast, only 68.3% of the CVID patients seroconverted, with median titers of specific Ab being 83-fold lower than in HC. In fact, only 4/46 patients (8.6%) of patients who were seronegative at baseline reached the threshold for an optimal response (250 U/mL). Using the EUROclass definition, patients with either a reduced proportion, but not absolute counts, of switched memory B-cells or having an increased frequency of CD21^low^ B-cells generally generated poor vaccine responses. Overall, CVID-patients had reduced spike-specific IFN-γ positive CD4^+^ T cell responses 2 weeks after the second dose, compared to HC. The total CD4 and CD4 central memory cell counts correlated with humoral immunity to the vaccine.

**Conclusions:**

CVID patients with low frequency of switched memory B-cells or an increased frequency of CD21^low^ B-cells according to the EUROclass definition demonstrated poor responses to Pfizer-BioNTech BNT162b2 mRNA vaccination. Cellular immune responses were significantly affected, affirming that the defect in CVID is not limited to humoral immunity.

**Supplementary Information:**

The online version contains supplementary material available at 10.1007/s10875-022-01244-2.

## Introduction

Common variable immunodeficiency disorders (CVID) form a heterogeneous group of immunodeficiencies likely encompassing several different etiologies [1]. Humoral immunity is affected in all patients, whereas defects in cellular immunity vary [2]. As pointed out in the *International Consensus Document (ICON)* on CVID, although there is considerable knowledge regarding the immunological response to a few vaccines, for most of them, information is scarce or lacking [3]. To this end, a rather unique feature of CVID is that vaccination is part of the diagnostic strategy. Common vaccines used include tetanus and diphtheria toxoids and antigens from *Haemophilus influenza* type B, and *Streptococcus pneumoniae.* The toxoids are proteins, whereas the bacterial vaccines are either polysaccharide-based or manufactured as polysaccharides conjugated to proteins. The majority of patients with CVID respond poorly to pure polysaccharide-vaccines but may mount weak responses to protein and conjugate vaccines [4]. While such vaccinations serve two purposes, diagnostic and prophylactic, the response may be difficult to interpret due to previous exposure to these bacteria in the past as well as previous vaccinations.

An important consideration is also the fact that CVID patients regularly are on immunoglobulin replacement therapy (IGRT). This means that for almost all infectious agents, there is a background of specific antibody (Ab) levels in the commercial immunoglobulin preparations, which makes it almost impossible to study primary immune responses without confounding contributions from the IGRT. Given these known problems with available vaccines, all SARS-CoV-2 vaccines are exceptional since the antigens used are new. Furthermore, it is known from the analysis of anti-spike IgG antibodies in the immunoglobulin preparations used at the time of the study that there were no detectable levels present [5–7].

Patients with CVID exhibit a variable susceptibility to Coronavirus disease 2019 (COVID-19), where those with underlying lung diseases, such as bronchiectasis and interstitial lung disease, have the highest risk [8]. In contrast, there are several case series that report a more moderate risk [9, 10]. However, the question of immune responses following COVID-19 vaccination in patients with CVID remains largely unanswered. We recently completed a large clinical trial with a prospective design, in which 449 patients with different immunocompromising disorders and 90 controls were vaccinated with the Pfizer-BioNTech BNT162b2 mRNA vaccine. In total, 50 patients with CVID were included in the study and 68.3% seroconverted [11], which is in line with a previous study from Israel (*n*=12 patients with CVID) [12]. Notably, a more recent and larger study from Italy could only detect spike-specific IgG in 20% of seronegative COVID-19 vaccinated patients with CVID (*n*=41). It was found that patients with a previous history of COVID-19 infection had higher titers after vaccination, suggesting that a natural infection may cause a more efficient priming of the immune system than the first vaccine dose [13]. Another study from Italy with 14 patients with CVID showed that most patients seroconverted after COVID-19-vaccination, but with lower titers than in healthy controls (HC) [14]. Hence, previous studies illustrate the fact that vaccine responses in patients with CVID are highly variable. Importantly, there is limited knowledge about the relation between immunological parameters and vaccine responses. Thus, we hypothesized that specific cellular subsets that are used to define CVID subgroups, including the EUROclass consensus definition, could correlate with outcome of COVID-19 vaccine-induced immune responses in patients with CVID [15]. To test this, we studied clinical and immunological data from patients with CVID, who were included in our recently completed COVID-19 vaccine trial [11], and correlated the results with seroconversion rates and spike IgG titers at day 35 (2 weeks after the second dose). Notably, the present study revealed that patients with an increased frequency of CD21^low^ B-cells had a significantly higher risk of seroconversion failure.

## Material and Methods

### Study Design and Participants

Details on the study design, protocol, and outcomes can be found in [11]. Briefly, the original study was conducted as an open-label, nonrandomized prospective clinical trial. The aim was to study the safety and efficacy of two doses of the Pfizer-BioNTech BNT162b2 mRNA (Comirnaty®, Pfizer/BioNTech) vaccine in immunocompromised patients and HC. In total, 539 patients were included, including 90 patients with primary immunodeficiency (PID), where 50 had CVID (presented in this follow-up study). The control group (*n*=90) consisted of individuals without an immunocompromised disorder or treatment and without significant comorbidity. The controls were selected to represent three age groups each of which included 30 healthy individuals (18–39 years, 40–59 years, and >60 years, respectively). The study was performed at the Karolinska University Hospital, Stockholm, Sweden. The study started recruiting on Feb 15, 2021, and follow-up is ongoing. Patients with a known diagnosis of previous or ongoing infection with SARS-CoV-2 assessed through patient interviews at screening were not included. Characterization of cellular subsets was performed at Clinical Immunology and Transfusion medicine, Karolinska University Hospital Laboratory, Huddinge, Sweden. T-cells were analyzed using a FITMAN standardized phenotyping panel developed by the Human Immunophenotyping Consortium (HIPC) [16], and for B-cells, a panel based on [17] was used. The gating strategies are outlined in Supplementary figure 1 (B-cell subsets) and Supplementary figure 2 (T-cell subsets).

### Regulatory and Ethical Approval and Written Informed Consent

The original study was approved by the Swedish Medical Product Agency (ID 5.1-2021-5881) and the Swedish Ethical Review Authority (ID 2021-00451). The trial was registered at EudraCT (no. 2021-000175-37) and clinicaltrials.gov (no. 2021-000175-37). All participants provided written informed consent prior to inclusion.

### Procedures

The participants were given injections of the Pfizer-BioNTech BNT162b2 mRNA vaccine in standard dose (30 micrograms) into the deltoid muscle of the nondominant arm on days 0 and 21 of the study, i.e., in a two-dose regimen according to the label. All vaccine doses were derived from the same batch (batch number EP2163). Blood samples were taken at day 0 (before the first vaccination) and then at days 10, 21 (before the second vaccination), and 35 (analysis of the primary endpoint).

### Antibody Test

Serum samples were tested using the commercial, quantitative Roche Elecsys anti-SARS-CoV-2 S enzyme immunoassay, which measures total antibodies to the SARS-CoV-2 spike receptor-binding domain (RBD) protein, the target of the mRNA vaccines. The results range from <0.4 to >250 U/mL with the positive cut-off defined as ≥0.80 U/mL, as specified from the manufacturer, and validated in one study [18]. Positive samples with ab titers of >250 U/mL were retested following a 1/10 dilution, and in applicable cases, also a 1/100 dilution which increased the upper level of measuring range to 25,000 U/mL.

### ELIspot Analysis

Cryopreserved PBMCs were thawed and rested for 3–4 h prior to assay. All experiments were seeded with 2.5 × 10^5^ PBMCs per well together with co-stimulatory anti-CD28/CD49d (347690, BD Biosciences) at 3 μL/mL and stimulated with either spike glycoprotein peptide pool (peptide & elephants, Germany) at 0.5 μg/mL or equivalent volume of DMSO (<1%). The assay was performed according to the manufacturer’s protocol using plates and reagents from a human IFN-γ ELISpot^PRO^ kit (3420-2APT-2, Mabtech). PBMCs were stimulated for 20 h in assay plates incubated at 37 °C and 5% CO_2_. Spots were counted with the IRIS ELISpot reader (Mabtech) and Apex software (Mabtech) using default settings. Visible artifacts were removed by masking. The results are expressed as spot forming units (SFU/10^6^ PBMCs) per 10^6^ cells, calculated by subtracting the mean of negative control wells from the mean of spike stimulation wells. A positive response after subtraction was determined by the mean of negative control wells plus two standard deviations (55 SFU/10^6^ PBMCs). To exclude cross-reactive responses, results were excluded from analysis if the negative control wells had >100 SFU/10^6^ PBMCs PBMCs or if the sample was positive (>55 SFU/10^6^ PBMCs) at baseline.

### Flow Cytometry (Activation-Induced Marker Assay)

Cryopreserved PBMCs were thawed quickly, resuspended in complete medium in the presence of DNase I (10 U/mL; Sigma-Aldrich), and rested at 1× 10^6^ cells/well in 96-well U-bottom plates (Corning) for 3 h at 37 °C. The medium was subsequently supplemented with anti-CXCR5–BB515 (clone RF8B2; BD Biosciences), followed 15 min later by the spike peptide pool (0.5 μg/mL), and a further 1 h later by brefeldin A(1 μg/ml; Sigma-Aldrich), monensin (0.7 μg/mL; BD Biosciences), and anti-CD107a–BV785 (clone H4A3; BioLegend). Negative control wells contained equivalent DMSO. After 9 h, cells were washed in PBS supplemented with 2% FBS and 2 mM EDTA (FACS buffer) and stained with anti-CCR4–BB700 (clone 1G1; BD Biosciences), anti-CCR6–BUV737 (clone 11A9; BD Biosciences), anti-CCR7–APC-Cy7 (clone G043H7; Bio-Legend), and anti-CXCR3–BV750 (clone 1C6; BD Biosciences) for 10 min at 37 °C. Additional surface stains were performed for 30 min at room temperature in the presence of Brilliant Stain Buffer Plus (BD Biosciences). Viable cells were identified by exclusion using a LIVE/DEAD Fixable Aqua Dead Cell Stain Kit (Thermo Fisher Scientific). Cells were then washed in FACS buffer and fixed/permeabilized using a FoxP3 Transcription Factor Staining Buffer Set (Thermo Fisher Scientific). Intracellular stains were performed for 30 min at room temperature. Stained cells were washed in FACS buffer, fixed in PBS containing 1% paraformaldehyde (PFA; Biotium), and acquired using a FACSymphony A5 (BD Biosciences). Flow cytometry panel and reagents are listed in Supplementary Table 1. 

## Statistical Analysis

Differences between groups were calculated using the Mann-Whitney *U* test for data with a skewed distribution. The Spearman rank correlation test was used to test for correlation between two parameters. Fisher’s exact test was used to test for statistical difference between ratios. GraphPadPrism 9.1.1 was used to make the figures and statistical tests. A *p* value of <0.05 was considered statistically significant.

## Results

### Study Cohort and Baseline Characteristics

The patients with CVID studied here represent a subset of an ongoing prospective, open-label clinical trial where 449 immunocompromised patients and 90 controls were vaccinated with two doses of the Pfizer-BioNTech BNT162b2 mRNA vaccine [11] and evaluated at day 35 (2 weeks after dose 2), as specified in the original safety and immunogenicity study [19]. In total, 50 patients with CVID were included in the present study. Four patients were excluded from final analyses due to the lack of samples on day 35. Complete data from 46 patients with CVID was analyzed, including 4 patients who were positive for anti-spike IgG antibodies at baseline and 1 who got COVID-19 during the study. These 5 patients were excluded from the *per protocol* analysis performed on patients with CVID (*n*=41) in the main paper (Table 4 in [11]). Likewise, five samples were lost to follow-up at day 35 in the control group leaving complete data from 85 controls. The patients with CVID and controls were roughly age- and sex-matched (*p*=0.69, Table 1).

**Table 1:** Baseline characteristics and titers for all CVID patients and healthy controls after 2 mRNA vaccine doses (day 35)

	CVID	Controls
Numbers in analysis (*n*)	46	85
Age (mean, +/− SEM)	50.5+/−2.2	51.0+/−1.9
Women (*n*, %)	26 (57%)	48 (56%)
Seroconversion (*n*, %)	33 (72%)	85 (100%)
Median titer d35 in units/mL (U)	26.6	2217
Interquartile range	0.4–146.8	1415–3916
Non-responders (<0.4 U, *n*, %)	13 (28%)	0 (0%)
Intermediate responders (0.4–250 U, *n*, %)	25 (54%)	0 (0%)
Optimal responders (>250 U, *n*, %)	8 (17%)	85 (100%)

### Lower Proportion of Seroconversion and Lower Antibody Titers in CVID Patients Versus Healthy Controls

**Fig. 1:**
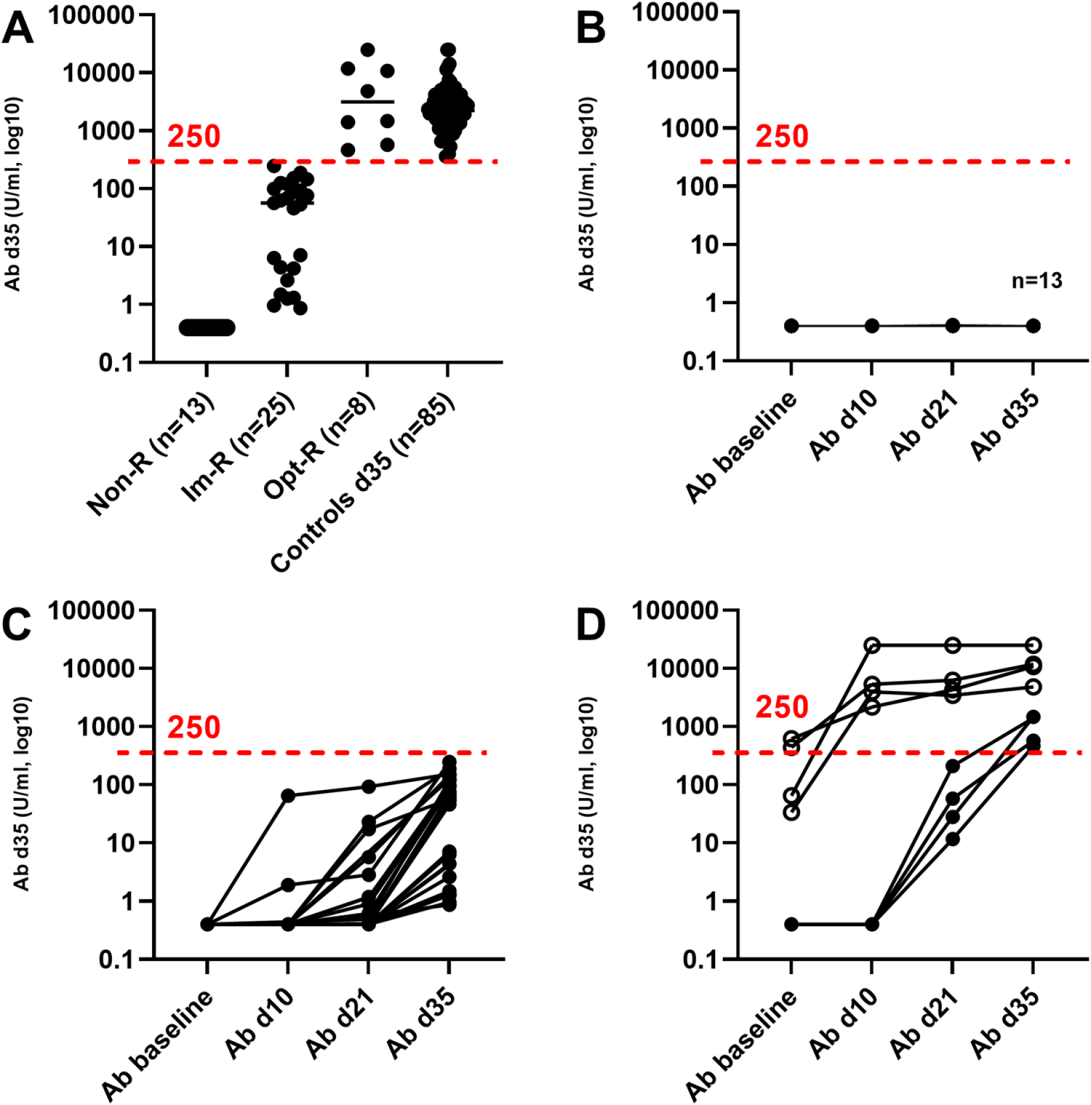
Antibody titers and kinetics in the CVID group compared to healthy controls. **A** Ab levels for all CVID patients and controls at day 35. The CVID group was divided into 3 subgroups based on their Ab response at day 35: nonresponders (Non-R, <0.4 U/ml), intermediate responders (IM-R, >0.4–250 U/ml), and optimal responders (Opt-R, >250 U/ml). **B**–**D** Kinetics of the antibody response in CVID patients and controls at baseline, d10, d21, and d35. Black line designates the median value of each column. (B) In the nonresponder group (*n*=13), none reached above 0.4 U/ml. **C** In the intermediate response group (*n*=25), all patients seroconverted, but none reached the optimal level of 250 U/ml at day 35. **D** In the optimal response group (*n*=8), the highest titers were observed in the those who were seropositive at baseline (*n*=4, open circles), whereas only *n*=4/42 who were seronegative at baseline reached 250 U/ml at day 35. Red-dotted lines designate the optimal antibody level, defined as the lowest level found in HC

Overall, 68.3% of the patients with CVID (28/41) who were seronegative at baseline and who did not have COVID-19 during the study seroconverted, as defined by having anti-spike IgG ≥0.8 Units/mL (U) after two vaccine doses, in comparison to the control group where 100% of the participants seroconverted. The proportion was slightly higher (33/46, 72%), when including patients being seropositive at baseline (*n*=4) and one patient who got COVID-19 during the study. Notably, the median Ab titer at day 35 was significantly lower (83-fold) in the CVID-group compared to that in the controls; 26.6 U (IQR 0.4–146.8 U) versus 2217 U (IQR 1415–3916 U), *p*<0.01, Table 1). Only 8/46 (17%) of the patients with CVID reached the level of 250 U after vaccination (median 3140, IQR 781–11546). A total of 25 patients had a value between 0.8 and 250 U/ml (median 56.7, IQR 3.4–109.4), and 13 patients did not respond to the vaccine (≥0.8 U/ml after two doses) (Table 1, Figure 1). Based on the vaccine response, the CVID group was further divided into three sub-groups: nonresponders (<0.8 U/ml), intermediate responders (≥0.8–250 U/ml), and optimal responders (>250 U/ml). As expected, the kinetic analysis showed that the best responders at day 35 were seropositive at baseline (*n*=4), all four reaching well above 250 U/ml already at day 10 after receiving their first dose, whereas very few (4/46, 9%) of the seronegative patients at baseline reached the Ab level of 250 U/ml. The single patient who contracted COVID-19 directly after the first dose seroconverted with a value of 152 U/ml (Figure 1D).

Next, we set out to assess relevant factors that could predict seroconversion in the CVID group. Notably, the high responders (Ab levels >250 U/ml) were younger than the non- and intermediate responders (*p*=0.05). In addition, the high responders had fewer concomitant diseases (comorbidity, lung disease, and autoimmunity, as outlined in Table 2) as well as fewer immunosuppressive medications, although the differences did not reach statistical significance (Table 2).

**Table 2:** Baseline characteristics for the 3 CVID groups

	Nonresp. (<0.4)	Im resp. (0.4–250)	Opt. resp. (>250)	*p* value
Number (*n*)	13	25	8	
Age (mean+/- SEM)	53.4+/−4.7	52.4+/−2.3	39.8+/−5.9	0.05**
Female (*n*, %)	6 (46%)	17 (68%)	3 (38%)	0.26*
Genetic diagn. (*n*, %)	0 (0%)	3 (12%)^1^	0 (0%)	0.99*
Co-morbidity (*n*, %)	4 (31%)^2^	10 (40%)^3^	1 (13%)^4^	0.24*
Lung disease (*n*, %)	5 (46%)^5^	8 (32%)^6^	0 (0%)	0.08*
Autoimmunity (*n*, %)	2 (15%)^7^	10 (40%)^8^	1 (13%)^9^	0.40*
IGRT (*n*, %)	13 (100%)	23 (92%)	8 (100%)	0.99*
Steroids (*n*, %)	2 (15%)	2 (15%)	0 (0%)	0.99*
Other Immunosuppr. (*n*, %)	3 (23%)^10^	2 (8%)^11^	0 (0%)	0.99*
Rituximab (*n*, %)	2 (15%)	0 (0%)	0 (0%)	0.99*

^1^Heterozygous TNFRSF13B-mutation (*n*=2), 15q24-deletion syndrome. ^2^GLILD (*n*=2), inflammatory diseases (*n*=2). ^3^Cryptogenic liver cirrhosis, chronic enteroviral shedding in the faeces, autoimmune neutropenia, M-component, GLILD, lymphoproliferation, polycytemia vera, diabetes type 1, diabetes type 2, epilepsy. ^4^Post status nefroblastoma. ^5^COPD, bronchiectasia and GLILD (*n*=3). ^6^Bronchiectasis (*n*=4), GLILD, COPD, granuloma in the lung and liver, interstitial lung disease. ^7^Lichen, rheumatoid arthritis. ^8^Hypothyreosis (*n*=2), autoimmune neutropenia, psoriasis (*n*=2), autoimmune hepatitis, autoimmune hemolysis, atrophic gastritis, mixed connective tissue disease, ITP. ^9^Gastrointestinal inflammation. ^10^Plaquenil, Imurel, and anti-TNF treatment and ^11^Ciklosporin and Stelara (anti-IL12/IL23 Ab). The statistical tests were performed between Non-R + Im-R (green shading) versus Opt-R (orange shading) using *Fisher’s exact test, except for age, where **Mann-Whitney *U* test was used. IGRT, immunoglobulin replacement therapy

### *Elevated Frequency of CD21*^*low*^* B-cells Correlates with Poor Vaccine-Induced Immunity*

**Fig. 2:**
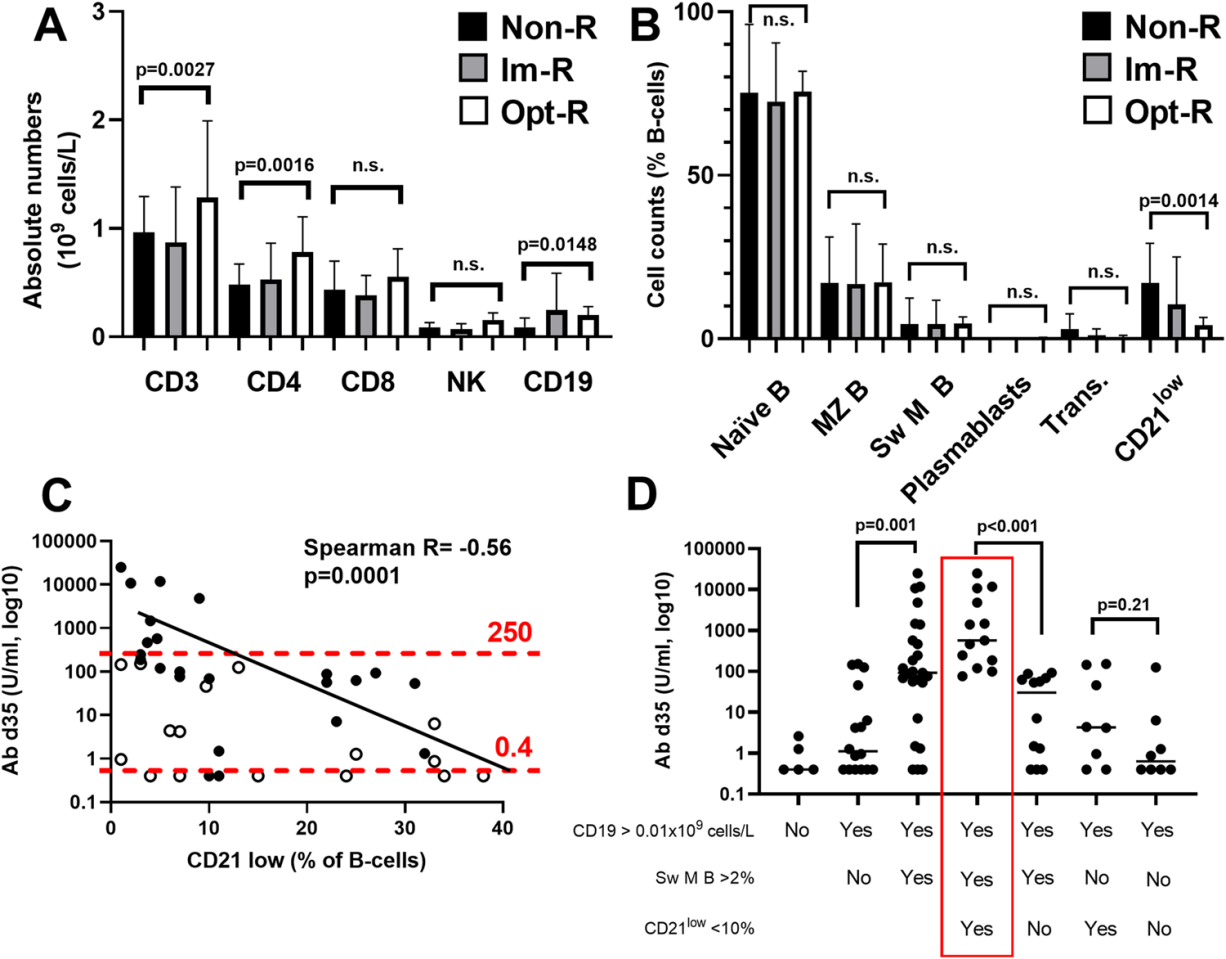
Lymphocyte profile and B-cell subsets for CVID patients divided in 3 groups based on their vaccine response. **A** The absolute numbers of CD3^+^, CD4^+^, CD8^+^, NK-, and CD19^+^ cells were defined by FACS analysis. Median values + interquartile ranges are plotted. **B** B-cell subsets were analyzed by FACS and plotted as % of the total number of CD19^+^ B-lymphocytes. Statistical significance was tested by Mann-Whitney *U* test. Non-R (*n*=13): nonresponders; Im-R (*n*=25), intermediate responders; and Opt-R (*n*=8), optimal responders. Median values + interquartile range is plotted. **C** Spearman correlation analysis of antibody titers (U/ml, log 10 units) and CD21^low^ (% of CD19^+^ B-cells). Red-dotted lines signify the cutoff levels for the 3 different response groups: nonresponders (<0.4 U/ml), intermediate responders (>0.4–250 U/ml), and optimal responders (>250 U/ml). The black line is an estimated correlation curve based on the *R* value of −0.56 as determined by the Spearman test. Open black circles designate patients with switched memory B-cells <2%. **D** Vaccine response in different CVID EUROclass groups. CD19, CD19^+^ B-cells; Sw M B, switched memory B-cells; CD21^low^, CD21^low^ B-cells. Statistical significance was tested by the nonparametric Mann-Whitney *U* test. The red box designates a group of optimal responders: CD19^+^-cells>0.01, Sw M B-cells >2%, and CD21^low^ < 10%. Black line designates the median value of each column.

Complete historical immunophenotyping data of T- and B-lymphocytes was obtained from the medical records of the patients and plotted for the three groups. Cellular counts revealed that nonresponders had lower counts of CD3^+^, CD4^+^, and CD19^+^ cells compared to the high responders (Figure 2A). Next, most B-cell subsets did not differ between the groups, except medians for CD21^low^ B-cell frequencies, which were significantly elevated in non- and intermediate responders at 11.0% (IQR 8.5–29) and 10.5% (IQR 5.3–25), whereas normal median levels (< 4%) were observed in optimal responders at 4.2% (IQR 2.4–4.9) (Figure 2B). Correlation analysis showed a clear negative association between an elevated frequency of CD21^low^ B-cells and a poor vaccine response (R= – 0.56; *p*<0.01, Figure 2C). Patients with a low proportion of switched memory B-cells (<2%) were generally poor responders, independent of CD21^low^ B-cell frequencies, and none reached 250 U (Figure 2C, open black circles). Notably, the absolute number of CD21^low^ B-cells did not correlate to the vaccine response, whereas the total numbers of naïve B-cells were significantly higher in the optimal responders (Suppl. figure 1).

### The Impact of the TNFRSF13B Mutation on the Vaccine Response

Interestingly, two of the patients in our cohort were genotyped and found to be carriers of a *TNFRSF13B* mutation in the heterozygous state (Table 2). On day 35, these patients had spike-specific Ab levels of 7.1 and 1.31 U, respectively. Thus, they seroconverted, but with a poor vaccine response. In addition, these patients had CD21^low^ B-cell frequencies of 23% and 32%, respectively (reference <4%).

### The EUROclass Definition of Patients with CVID Can Predict Optimal Responders

**Fig. 3:**
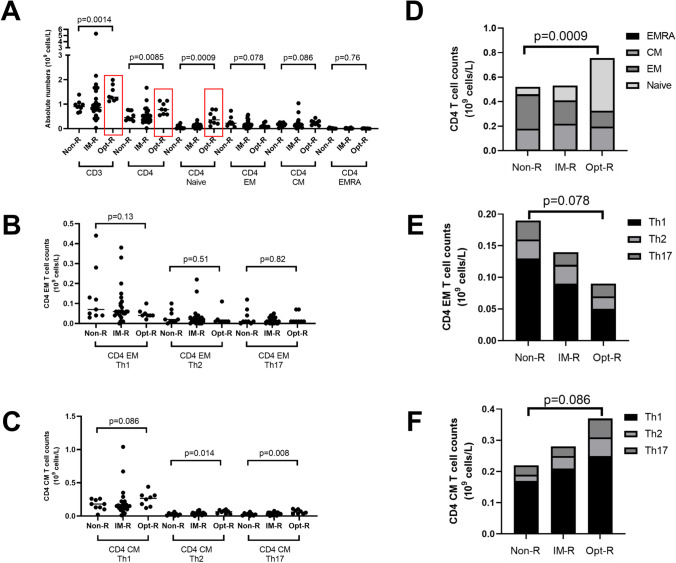
Analysis of absolute numbers of T-cells and relevant subsets in relation to the vaccine response. **A** Absolute numbers of T-cells in relation to nonresponders (non-R), intermediate responders (IM-R), and optimal responders (Opt-R), as defined in the materials and method section. Red boxes designate significant changes. **B** Absolute numbers of CD4^+^ effector memory (EM) subsets: Th1, Th2, and Th17 cells in relation to the vaccine response. **C** Absolute numbers of CD4^+^ central memory (CM) subsets: Th1, Th2, and Th17 cells in relation to the vaccine response. **D** Stacked data on absolute numbers of CD4^+^ T-cell subsets in relation to the vaccine response. **E** Stacked data on absolute numbers of CD4^+^ EM T-cell subsets in relation to the vaccine response. **F** Stacked data on absolute numbers of CD4^+^ CM T-cell subsets in relation to the vaccine response. The Mann-Whitney *U* test was used to test for statistical significance; *p*<0.05 was considered to be significant

In 2008, Wehr et al. proposed a consensus definition of different CVID subgroups based on B-cell phenotypic profiling, the EUROclass-definition [15]. We therefore divided the patients into the different EUROclass groups to assess patterns of responses (Figure 3D). First, as expected, it was apparent that B-cells were necessary for a vaccine response, since those with low or undetectable levels of CD19^+^ B-cells (B^-^ group, according to EUROclass) showed a very poor Ab response. Next, including only those with detectable levels of B-cells (B^+^-group), it is shown that those with >2% memory B-cells (smB^+^) had a significantly better Ab response compared to those with <2% (smB^-^) (Figure 2D, p<0.01). Further, when the smB^+^ group was divided based on the frequencies of CD21^low^-cells, it was clearly seen that those with normal CD21^low^ B-cells (<10%, smB^+^, CD21^norm^) had a significantly better Ab response than those with an increased percentage of CD21^low^ B-cells (<10%, smB^+^, CD21^low^). Finally, the frequency of CD21^low^ cells did not impact the Ab response in patients with switched memory B-cells <2% (Figure 2D). Thus, all patients with detectable B-cells, switched memory B-cells >2%, and normal frequencies of CD21^low^ B-cells seroconverted, underscoring the importance of particular B-cell subsets for a proper Ab response to mRNA vaccination.

### *Reduced Levels of Naïve CD4*^*+*^* T-cells Correlate with a Poor Vaccine Response*

Next, we compiled data on absolute counts of T-cell subsets and related them to the vaccine response. Overall, reduced numbers of CD4^+^, but not CD8^+^, T-cells correlated with a poor vaccine response (Figures 2A and 3A). The CD4^+^ T-cell pool was further divided into naïve, effector memory (EM), central memory (CM), and effector memory cells re-expressing CD45RA (EMRA). Reduced levels of naïve CD4^+^ T-cells were associated with a poor vaccine response (Figure 3A and D). Next, the memory subsets were further subdivided into Th1, Th2, and Th17 cells (Figure 3B and C). Notably, no statistically significant differences were found for the EM subset, whereas for the CM subset, reduced levels of Th2 and Th17 cells were associated with a poor vaccine response (Figure 3B and C). Finally, a poor vaccine response was weakly associated with elevated levels of effector memory cells and reduced central memory cells, although the difference did not reach statistical significance (Fig. 3E and F).

### *Lower CD4*^*+*^* T-cell Responses in the CVID-Group Versus Healthy Controls*

**Fig. 4:**
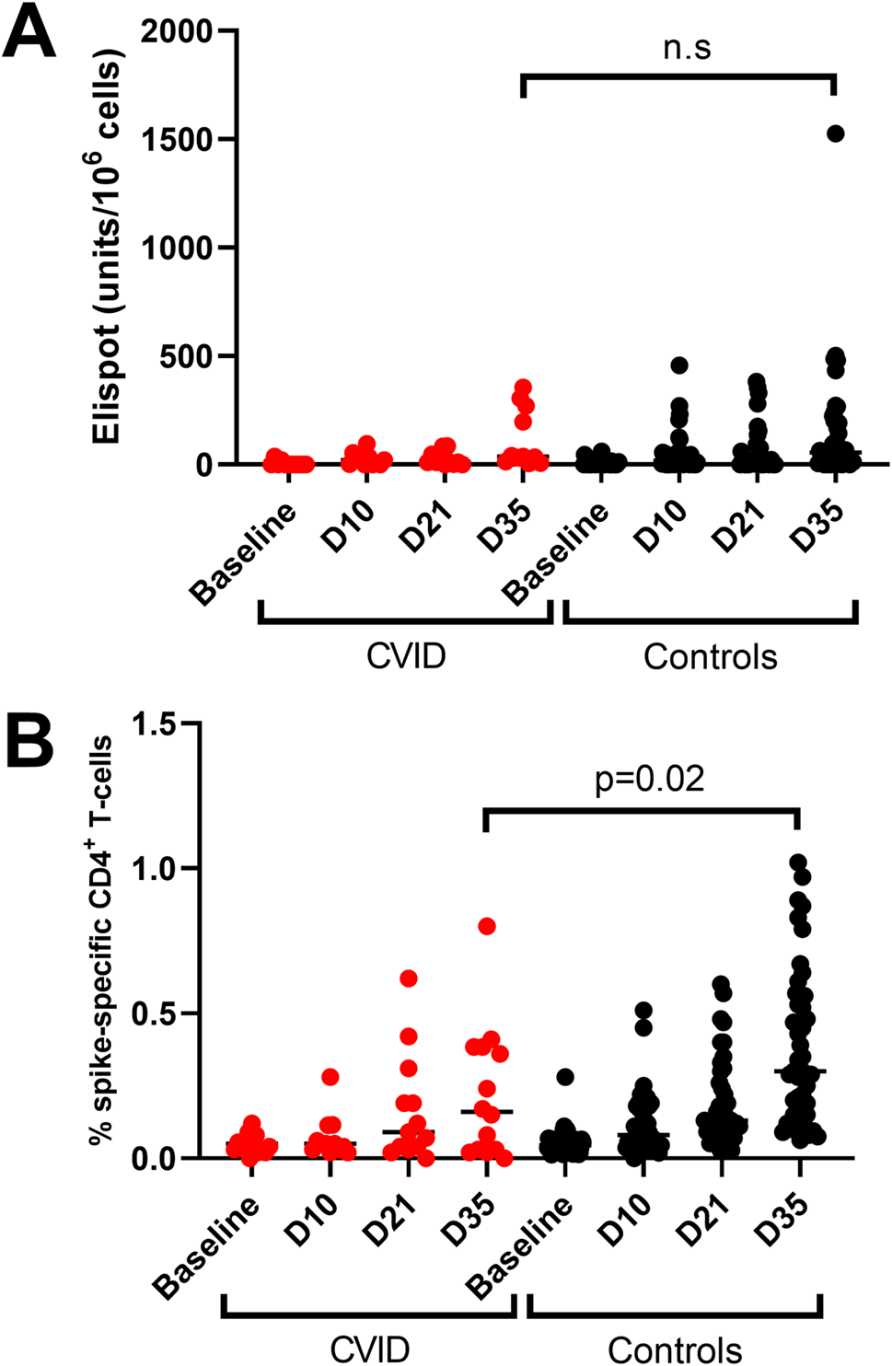
Spike IFN-γ and CD4^+^ T-cell responses in patients with CVID and controls. **A** ELIspot responses for patients with CVID (*n*=11, red dots) and controls (*n*=45, black dots) are shown as a dot-plot. Median values are marked with horizontal lines. There was no statistically significant difference between median spot forming units (SFUs) at day 35 between patients with CVID and healthy controls (*p*=0.77, Mann-Whitney *U* test). **B** Spike-specific CD69^+^, CD154^+^, and CD4^+^ T-cell responses for patients with CVID (*n*=16) and controls (*n*=44), plotted as % of CD4^+^ T-cells. Patients with CVID had a lower spike-specific CD69^+^, CD154^+^, CD4^+^ T-cell response at day 35, compared to healthy controls (*p*=0.02, Mann-Whitney *U* test)

PBMCs were collected from a subset of the patients with CVID and used to study T-cell responses with ELIspot and FACS. Using ELIspot, patients with CVID produced significantly higher amounts of spike-specific IFN-γ secreting cells after two vaccine doses at day 35 compared to baseline (*p*<0.01, Mann-Whitney *U* test). There was no difference in median SFU between patients (median 38 SFU, IQR 14–270 SFU) and HC (median 56 SFU, IQR 11–201) at day 35 (*p*=0.77, Mann-Whitney *U* test) (Figure 4A). IFN-γ positive cells (>55 SFU) were detected in 4/11 (36.4%) patients across all subgroups, which was not significantly different from HC, where 20/35 (57.1%) had IFN-γ positive cells (>55 SFU, *p*=0.31, Fisher’s exact test) (Figure 4A). Next, spike-specific CD4^+^ T cell responses were analyzed by using activation-induced markers (CD69 and CD154) in FACS. In this analysis, 9/14 (64%) patients with CVID responded above the cutoff (>0.05%) at day 35, compared to 44/44 (100%) HC (*p*<0.01, Fisher’s exact test) (Figure 4B). Moreover, the *magnitude* of spike-specific CD4^+^ T cell responses was lower for patients with CVID (median 0.16%, IQR 0.035–0.25%) compared to HC (median 0.30%, IQR 0.15–0.56%), *p*=0.02, Mann-Whitney *U* test (Figure 4B).

## Discussion

The main finding in this report was the observation that a high frequency of the CD21^low^ B-cell subset significantly correlated with a poor primary humoral immune response. While this marker previously has been used to identify subsets of patients with CVID [15, 20–22], to the best of our knowledge, this is the first time that the frequency of CD21^low^ B-cells was found to significantly correlate with the capacity to mount a humoral immune response to a true neoantigen.

Additionally, the EUROclass consensus definition clearly defined a group of responders to vaccination. Thus, all patients with CVID having detectable levels of B-cells switched memory B-cells >2% and a normal percentage of CD21^low^ B-cells seroconverted in this study, whereas those patients with either switched memory B-cells <2% or an increased frequency of CD21^low^ B-cells (>10%) exhibited a poor vaccine response. This finding confirms that the EUROclass definition can be useful in clinical practice for identifying patients at risk for a poor response to a protein antigen expressed in the form of an mRNA vaccine.

Next, the CD4^+^ T-cell phenotypic profiles of the patients were analyzed and specific cellular subsets were related to the vaccine response. Notably, reduced levels of CD4^+^, but not CD8^+^, T-cells were associated with a poor vaccine response. In the CD4^+^ T-cell pool, mainly elevated levels of naïve CD4^+^ T-cells (CD45RA+, CCR7+) corresponded to an optimal vaccine response. Interestingly, patients with a poor vaccine response had lower levels of central memory (CM) cells, particularly CM Th2 and Th17 cells (Figure 3F). These findings suggest that the subgroup of CVID-patients with reduced levels of specific T-cell subsets, previously connected to autoimmune complications, also may suffer from a poor vaccine response to mRNA vaccination [21, 23].

We also assessed the cellular immune response in a subset of patients with CVID, using an ELIspot IFN-γ release assay as well as a spike-specific AIM CD4 T-cell assay (FACS). Overall, our results show that patients with CVID exhibited poor cellular immunity, with only a fraction of patients reaching the same response as HC. This indicates that patients with CVID have a combined deficiency in both the cellular and humoral immune response to mRNA vaccination.

The observed increased frequency of the CD21^low^ B-cells among nonresponders to COVID-19 vaccination is of interest since this population has been implicated in immune responses. Thus, CD21^low^ B-cells have been suggested to represent a distinct population, which is transiently induced 2 to 4 weeks after immunization and recently egressed from germinal centers [24]. While carrying CD27, they are not classical memory cells and express high levels of the transcription factor T-bet [24–26]. T-bet–expressing cells are also induced by chronic viral infections, including HIV and hepatitis C [27, 28]. CD21^low^ B-cells are also implicated in autoimmunity, such as in systemic lupus erythematosus (SLE)[29, 30]. This raises the question whether CD21^low^ B-cells are *directly* implicated in the poor vaccine response or if they merely serve as a *marker* of a subnormal response.

Since the frequency, but not the absolute number, of CD21^low^ B-cells correlates with vaccine-induced immunity (Suppl fig. 1), we favor the idea that CD21^low^ is a useful marker but that this subset only indirectly contributes to poor immunity. In line with this reasoning, it was previously reported that the increased proportion of CD21^low^ cells in CVID is the indirect result of reduced naïve B-cell numbers [31]. In fact, we also observed that reduced numbers of naïve B-cells were associated with a poor vaccine response (Suppl figure 1). Furthermore, it was recently found that CD21^low^ cells can serve as competent antigen-presenting cells in an allogeneic co-culture system [32], suggesting that an elevated proportion of these cells is not directly implicated in poor vaccine-mediated immunity.

Germinal center B-cells are necessary for the induction of potent humoral immunity against SARS CoV-2 mRNA vaccines [33]. Interestingly, patients with CVID may have dysfunctional and irregular germinal centers, which are associated with an increased percentage of CD21^low^ B-cells in the circulation [34]. In addition, patients with CVID exhibit profound defects in the maturation of pre germinal center B-cells [35]. Thus, it is possible that an increased percentage of CD21^low^ B-cells could serve as a marker for dysfunctional germinal center reactions in CVID patients and effectively predict nonresponders to mRNA vaccination.

This study has several strengths. First, the data were collected from a bona fide prospective clinical trial with the scientific and regulatory rigor inherent to this design, which contributed to high data quality, low dropout rates and minimal risk of selection bias. Second, by studying the immune reaction to a neoantigen and thereby avoiding problems with pre-existing immunity, this study, as well as others studying SARS-CoV-2 responses, is likely unique [36]. Thus, while the effect of influenzae vaccination has been investigated in patients with CVID [37], pre-existing immunity makes it difficult to differentiate between primary and recall responses. Finally, the study is one of the largest studies on patients with CVID in relation to COVID-19 vaccination, which allowed subgroup analyses with enough power to obtain statistical significance and clinically meaningful outcomes. However, certain subgroups were too small to allow for robust analyses. Thus, the low immune responses noted in two patients with TNFRSF13B-mutations will need confirmation in larger follow-up studies with more individuals, including both heterozygous and homozygous carriers.

Despite these strengths, several weaknesses need to be acknowledged. First, the T- and B-cell data was collected up to 4 years prior to the vaccine study, which could lead to lower precision in the analyses. However, the immunological aberrations in patients with CVID do not fluctuate much but rather remain over time [15]. For example, when checking our own records, where available, most individuals with an increased frequency of CD21^low^ B-cells sustain this immunological phenotype over at least 5 years (data not shown). Thus, despite this potential confounder, our data are valid and clinically relevant. Further, due to logistical constraints, we could only collect PBMCs from a subset of patients, which makes it difficult to draw strong conclusions on T-cell immunity from subgroup analyses. Another limitation is the lack of mechanistic insight for the observed influence of the percentage of CD21^low^-B cells and how this can lead to a poor immune response. While there has been considerable development in the understanding of this subset, many aspects remain elusive [26]. At this point, we favor the idea that an elevated percentage of CD21^low^ B-cells is an indirect marker for disturbed B-cell–mediated immunity in patients with CVID.

A potentially interesting observation is the fact that in SLE, the CD21^low^ B-cell population has been suggested to represent an innate cell type related to TLR7 [29, 38]. Given the recent finding that 1.8% of men below the age of 60 who develop life-threatening COVID-19 have a defective TLR7 [39], there may also be other connections of importance for how CD21^low^ B-cells interact with SARS-CoV-2 vaccines. CD21^low^ B-cells have been shown to mount impaired calcium mobilization and NF-κB activation after B-cell receptor stimulation. This response could potentially be dysfunctional in a primary immune response against viral infection [40], but given the fact that the percentage, but not the absolute number, of CD21^low^ B-cells is increased, we do not favor this idea. Thus, the mechanistic understanding of vaccine responses in patients with CVID requires further studies, where especially the role of different T-cell subsets for the development of the humoral immunity is taken into account.

## Conclusions

The main finding presented here is that an increased percentage of CD21^low^ B-cells was associated with a poor vaccine response, suggesting that the EUROclass classification was useful to define a group of patients with CVID and an adequate vaccine response as well as poor responders. This information could have clinical implications, since it could facilitate the selection of patients with increased risk for seroconversion failure. Also demonstrated was that T-cell immunity to the vaccine was impaired and correlated to a poor humoral immune response to the vaccine in many patients with CVID. This, together with a weak humoral immune response, suggests that these patients will need careful attention and early treatment during the pandemic. A third vaccine dose, and possibly additional booster doses to maintain immunity, should be provided to all patients with CVID, and in case of breakthrough infections, monoclonal Ab therapy could be initiated to protect this vulnerable patient group against SARS CoV-2 infection and severe COVID-19.

## Supplementary Information

Below is the link to the electronic supplementary material.

Supplementary file1 (DOCX 975 KB)

## Data Availability

Data availability is specified in the original study [11].

## References

[1] International Union of Immunological Societies Expert Committee on Primary I, Notarangelo LD, Fischer A, Geha RS, Casanova JL, Chapel H*, et al.* Primary immunodeficiencies: 2009 update. J Allergy Clin Immunol. 2009;124(6):1161–1178. 10.1016/j.jaci.2009.10.013 PMC2797319 20004777

[2] Fernando SL, Jang HS, Li J (2021). The immune dysregulation of common variable immunodeficiency disorders. Immunol Lett..

[3] Bonilla FA, Barlan I, Chapel H, Costa-Carvalho BT, Cunningham-Rundles C, de la Morena MT (2016). International Consensus Document (ICON): common variable immunodeficiency disorders. J Allergy Clin Immunol Pract..

[4] Milito C, Soccodato V, Collalti G, Lanciarotta A, Bertozzi I, Rattazzi M*, *et al. Vaccination in PADs. Vaccines (Basel). 2021;9(6):626. 10.3390/vaccines9060626 PMC8230118 34207916

[5] Dalakas MC, Bitzogli K, Alexopoulos H (2021). Anti-SARS-CoV-2 antibodies within IVIg preparations: cross-reactivities with seasonal coronaviruses, natural autoimmunity, and therapeutic implications. Front Immunol..

[6] Karbiener M, Farcet MR, Schwaiger J, Powers N, Lenart J, Stewart JM, et al. Plasma from post-COVID-19 and COVID-19-vaccinated donors results in highly potent SARS-CoV-2 neutralization by intravenous immunoglobulins. J Infect Dis. 2021 Sep 20:jiab482. 10.1093/infdis/jiab482 PMC8499975 34543421

[7] Kubota-Koketsu R, Terada Y, Yunoki M, Sasaki T, Nakayama EE, Kamitani W (2021). Neutralizing and binding activities against SARS-CoV-1/2, MERS-CoV, and human coronaviruses 229E and OC43 by normal human intravenous immunoglobulin derived from healthy donors in Japan. Transfusion..

[8] Milito C, Soccodato V, Auria S, Pulvirenti F, Quinti I (2021). COVID-19 in complex common variable immunodeficiency patients affected by lung diseases. Curr Opin Allergy Clin Immunol..

[9] Drabe CH, Hansen AE, Rasmussen LD, Larsen OD, Moller A, Mogensen TH (2021). Low morbidity in Danish patients with common variable immunodeficiency disorder infected with severe acute respiratory syndrome coronavirus 2. Infect Dis (Lond)..

[10] Lindahl HS, CIE.; Bergman, P. COVID-19 in a patient with Good's syndrome and in 13 patients with common variable immunodeficiency. Clin Immunol Commun. 2021;1:20–24. 10.1016/j.clicom.2021.08.003 PMC8497938 38620775

[11] Bergman P, Blennow O, Hansson L, Mielke S, Nowak P, Chen P*, *et al. Safety and efficacy of the mRNA BNT162b2 vaccine against SARS-CoV-2 in five groups of immunocompromised patients and healthy controls in a prospective open-label clinical trial. EBioMedicine. 2021;74:103705. 10.1016/j.ebiom.2021.103705 PMC8629680 34861491

[12] Hagin D, Freund T, Navon M, Halperin T, Adir D, Marom R, et al. Immunogenicity of Pfizer-BioNTech COVID-19 vaccine in patients with inborn errors of immunity. J Allergy Clin Immunol . 2021 Sep;148(3):739-749. 10.1016/j.jaci.2021.05.029 PMC8168345 34087242

[13] Salinas AF, Mortari EP, Terreri S, Quintarelli C, Pulvirenti F, Di Cecca S (2021). SARS-CoV-2 vaccine induced atypical immune responses in antibody defects: everybody does their best. J Clin Immunol..

[14] Amodio D, Ruggiero A, Sgrulletti M, Pighi C, Cotugno N, Medri C*, *et al. Humoral and cellular response following vaccination with the BNT162b2 mRNA COVID-19 vaccine in patients affected by primary immunodeficiencies. Front Immunol. 2021;12:727850. 10.3389/fimmu.2021.727850 PMC8521226 34671350

[15] Wehr C, Kivioja T, Schmitt C, Ferry B, Witte T, Eren E (2008). The EUROclass trial: defining subgroups in common variable immunodeficiency. Blood..

[16] Maecker HT, McCoy JP, Nussenblatt R (2012). Standardizing immunophenotyping for the Human Immunology Project. Nat Rev Immunol..

[17] Warnatz K, Schlesier M (2008). Flowcytometric phenotyping of common variable immunodeficiency. Cytometry B Clin Cytom..

[18] Higgins V, Fabros A, Kulasingam V (2021). Quantitative measurement of anti-SARS-CoV-2 antibodies: analytical and clinical evaluation. J Clin Microbiol..

[19] Walsh EE, Frenck RW, Falsey AR, Kitchin N, Absalon J, Gurtman A (2020). Safety and immunogenicity of Two RNA-based covid-19 vaccine candidates. N Engl J Med..

[20] Rakhmanov M, Keller B, Gutenberger S, Foerster C, Hoenig M, Driessen G (2009). Circulating CD21low B cells in common variable immunodeficiency resemble tissue homing, innate-like B cells. Proc Natl Acad Sci U S A..

[21] Unger S, Seidl M, van Schouwenburg P, Rakhmanov M, Bulashevska A, Frede N (2018). The TH1 phenotype of follicular helper T cells indicates an IFN-gamma-associated immune dysregulation in patients with CD21low common variable immunodeficiency. J Allergy Clin Immunol..

[22] Warnatz K, Denz A, Drager R, Braun M, Groth C, Wolff-Vorbeck G (2002). Severe deficiency of switched memory B cells (CD27(+)IgM(-)IgD(-)) in subgroups of patients with common variable immunodeficiency: a new approach to classify a heterogeneous disease. Blood..

[23] Wiesik-Szewczyk E, Rutkowska E, Kwiecien I, Korzeniowska M, Soldacki D, Jahnz-Rozyk K (2021). Patients With common variable immunodeficiency complicated by autoimmune phenomena have lymphopenia and reduced Treg, Th17, and NK cells. J Clin Med..

[24] Lau D, Lan LY, Andrews SF, Henry C, Rojas KT, Neu KE*, *et al*.* Low CD21 expression defines a population of recent germinal center graduates primed for plasma cell differentiation. Sci Immunol. 2017;2(7):eaai8153. 10.1126/sciimmunol.aai8153 PMC5896567 28783670

[25] Johnson JL, Rosenthal RL, Knox JJ, Myles A, Naradikian MS, Madej J, et al. The transcription factor T-bet resolves memory B cell subsets with distinct tissue distributions and antibody specificities in mice and humans. Immunity. 2020;52(5):842–855 e6. 10.1016/j.immuni.2020.03.020 PMC7242168 32353250

[26] Keller B, Strohmeier V, Harder I, Unger S, Payne KJ, Andrieux G*, *et al*.* The expansion of human T-bet(high)CD21(low) B cells is T cell dependent. Sci Immunol. 2021;6(64):eabh0891. 10.1126/sciimmunol.abh0891 34623902

[27] Knox JJ, Buggert M, Kardava L, Seaton KE, Eller MA, Canaday DH*, *et al*.* T-bet+ B cells are induced by human viral infections and dominate the HIV gp140 response. JCI Insight. 2017;2(8):e92943. 10.1172/jci.insight.92943 PMC5396521 28422752

[28] Knox JJ, Kaplan DE, Betts MR (2017). T-bet-expressing B cells during HIV and HCV infections. Cell Immunol..

[29] Jenks SA, Cashman KS, Zumaquero E, Marigorta UM, Patel AV, Wang X*, et al.* Distinct effector B cells induced by unregulated toll-like receptor 7 contribute to pathogenic responses in systemic lupus erythematosus. Immunity. 2018;49(4):725–739 e6. 10.1016/j.immuni.2018.08.015 PMC6217820 30314758

[30] Tipton CM, Fucile CF, Darce J, Chida A, Ichikawa T, Gregoretti I (2015). Diversity, cellular origin and autoreactivity of antibody-secreting cell population expansions in acute systemic lupus erythematosus. Nat Immunol..

[31] Edwards ESJ, Bosco JJ, Aui PM, Stirling RG, Cameron PU, Chatelier J (2019). Predominantly antibody-deficient patients with Non-infectious complications have reduced naive B, Treg, Th17, and Tfh17 cells. Front Immunol..

[32] Reincke ME, Payne KJ, Harder I, Strohmeier V, Voll RE, Warnatz K, et al. The antigen presenting potential of CD21(low) B cells. Front Immunol. 2020;11:535784. 10.3389/fimmu.2020.535784 PMC7609862 33193306

[33] Turner JS, O'Halloran JA, Kalaidina E, Kim W, Schmitz AJ, Zhou JQ (2021). SARS-CoV-2 mRNA vaccines induce persistent human germinal centre responses. Nature..

[34] Unger S, Seidl M, Schmitt-Graeff A, Bohm J, Schrenk K, Wehr C (2014). Ill-defined germinal centers and severely reduced plasma cells are histological hallmarks of lymphadenopathy in patients with common variable immunodeficiency. J Clin Immunol..

[35] Del Pino-Molina L, Lopez-Granados E, Lecrevisse Q, Torres Canizales J, Perez-Andres M, Blanco E, et al. Dissection of the pre-germinal center B-cell maturation pathway in common variable immunodeficiency based on standardized flow cytometric EuroFlow tools. Front Immunol. 2020;11:603972. 10.3389/fimmu.2020.603972 PMC7925888 33679693

[36] Andrews SF, Chambers MJ, Schramm CA, Plyler J, Raab JE, Kanekiyo M, et al. Activation dynamics and immunoglobulin evolution of pre-existing and newly generated human memory B cell responses to influenza hemagglutinin. Immunity. 2019;51(2):398-410 e5. 10.1016/j.immuni.2019.06.024 31350180

[37] Gardulf A, Abolhassani H, Gustafson R, Eriksson LE, Hammarstrom L. Predictive markers for humoral influenza vaccine response in patients with common variable immunodeficiency. J Allergy Clin Immunol. 2018;142(6):1922–1931 e2. 10.1016/j.jaci.2018.02.052 29678747

[38] Zumaquero E, Stone SL, Scharer CD, Jenks SA, Nellore A, Mousseau B*, *et al*.* IFNgamma induces epigenetic programming of human T-bet(hi) B cells and promotes TLR7/8 and IL-21 induced differentiation. Elife. 2019;8. 10.7554/eLife.41641 PMC6544433 31090539

[39] Asano T, Boisson B, Onodi F, Matuozzo D, Moncada-Velez M, Maglorius Renkilaraj MRL*, *et al*.* X-linked recessive TLR7 deficiency in ~1% of men under 60 years old with life-threatening COVID-19. Sci Immunol. 2021;6(62):eabl4348. 10.1126/sciimmunol.abl4348 PMC8532080 34413140

[40] Freudenhammer M, Voll RE, Binder SC, Keller B, Warnatz K (2020). Naive- and memory-like CD21(low) B cell subsets share core phenotypic and signaling characteristics in systemic autoimmune disorders. J Immunol..

