# Supplementary figures and images for: Elevated CD21low B Cell Frequency Is a Marker of Poor Immunity to Pfizer-BioNTech BNT162b2 mRNA Vaccine Against SARS-CoV-2 in Patients with Common Variable Immunodeficiency
Source: J Clin Immunol. 2022 Mar 15;42(4):716–27. doi: 10.1007/s10875-022-01244-2 (PMC8922070; doi:10.1007/s10875-022-01244-2)

**Suppl. Fig 1**

**Suppl. Fig 2**


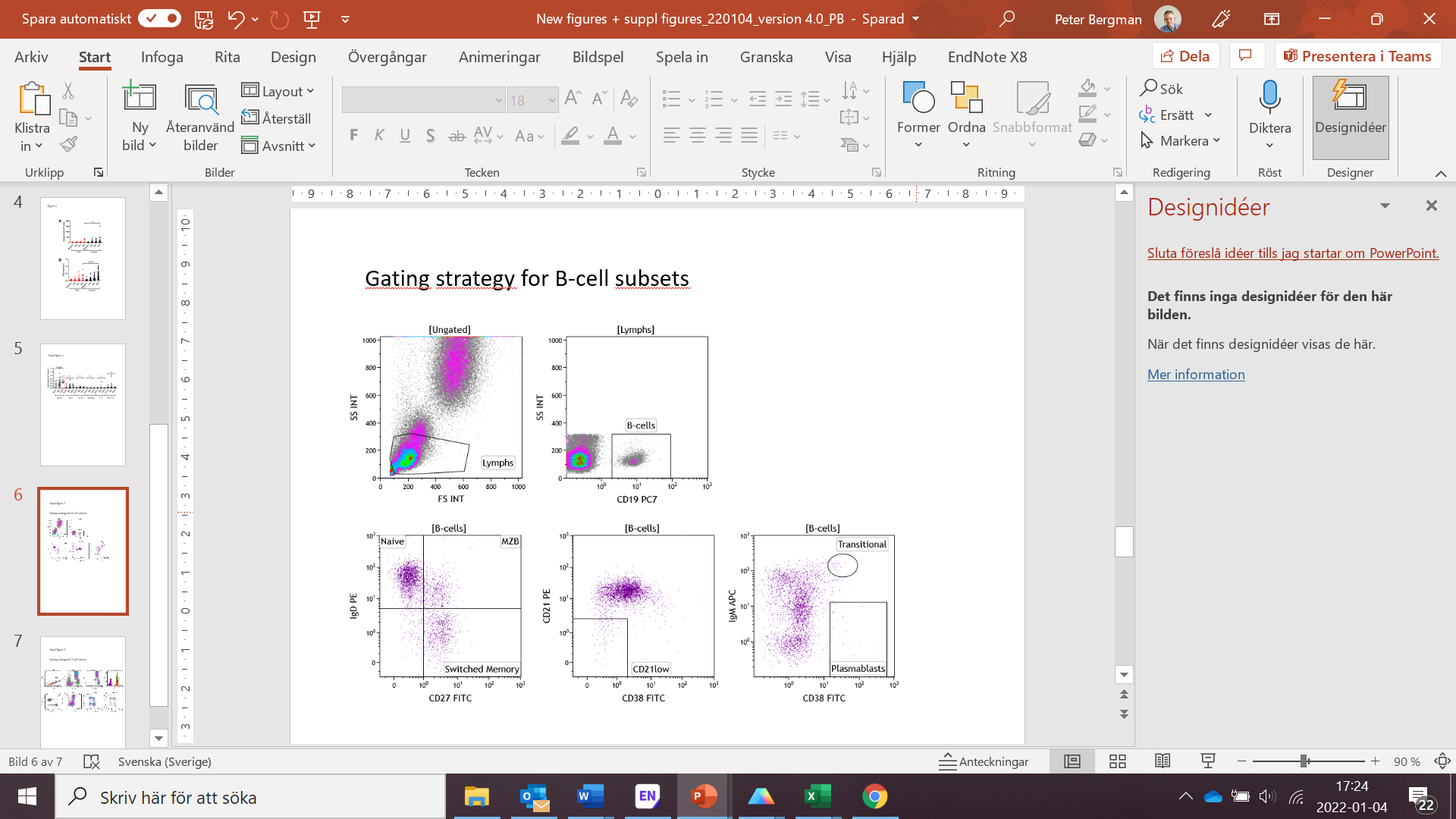


**Suppl. Fig 3**


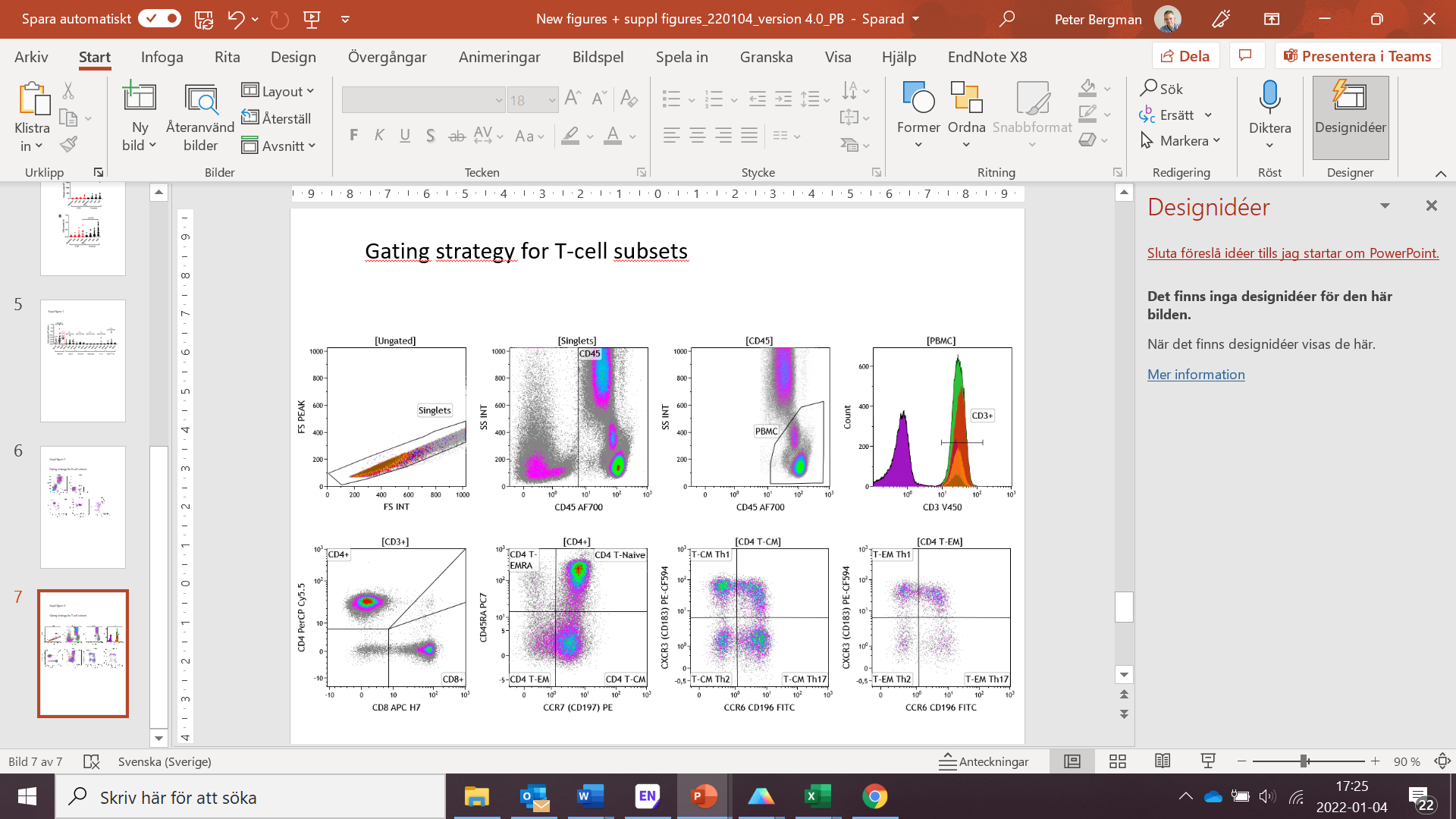

Supplement: Supplementary file 1 — Supplementary file1 (DOCX 975 KB) [file 10875_2022_1244_MOESM1_ESM.docx]
